# Long-term follow-up of a phase 1/2 trial of anti-GDF-15 antibody visugromab plus anti-PD-1 antibody nivolumab in anti-PD-1/-L1 relapsed/refractory solid tumors

**DOI:** 10.1186/s13045-026-01818-2

**Published:** 2026-07-09

**Authors:** Ignacio Melero, María de Miguel, Elena Garralda Cabanas, Guillermo de Velasco, Markus Joerger, Juan Martín-Liberal, Maria Reig, David König, Joerg Trojan, Maria-Elisabeth Goebeler, Martin Schuler, Guzman Alonso, Reinhard Dummer, Maria E. Rodríguez-Ruiz, Ramón Yarza, Giulia Pretelli, Jorge Esteban-Villarubia, Kira-Lee Koster, Paula Sabat Viltro, Marco Sanduzzi-Zamparelli, Heinz Läubli, Christine Koch, Cyrus Sayehli, Tanja Gromke, Fabricio Racca, Egle Ramelyte, Peter R. Galle, Andrea Necchi, Martin Reck, Zlatko Trajanoski, Hubert Hackl, Falk Gogolla, Jaclyn Billing, Teresa Sattmann, Jörg Wischhusen, Christine Schuberth-Wagner, Julia Akdemir, Felix S. Lichtenegger, Alexandra Auckenthaler, Marlene Fox, Kathrin Klar, Petra Fettes, Mara Liebig, Aasim Amin, Sheena Sachdeva, Frank Hermann, Eugen Leo

**Affiliations:** 1 Clinica Universidad de Navarra, CIMA, IDISNA and CIBERONC, PÍO XII Avenue 36, Pamplona, 31008 Spain; 2 Nuffield Department of Medicine, University of Oxford, Oxford, United Kingdom; 3 START Madrid-CIOCC, Centro Integral Oncologico Clara Campal, Madrid, Spain; 4 Vall d’Hebron Institute of Oncology (VHIO), Hospital Universitari Vall d’Hebron, Barcelona, Spain; 5 Medical Oncology Department, Hospital 12 de Octubre, Madrid, Spain; 6 Department of Medical Oncology & Hematology, Cantonal Hospital, St Gallen, Switzerland; 7 Medical Oncology Department, Catalan Institute of Oncology (ICO), L’Hospitalet de Llobregat, Barcelona, Spain; 8 Liver Oncology Unit, Liver Unit, BCLC group, Hospital Clinic of Barcelona, Barcelona, Spain; 9 Institut d’Investigacions Biomèdiques August Pi i Sunyer (IDIBAPS), Barcelona, Spain; 10 Centro de Investigacion Biomedica en Red en Enfermedades Hepaticas y Digestivas (CIBEREHD), Madrid, Spain; 11 Department of Medical Oncology, University Hospital Basel, Basel, Switzerland; 12 Medical Clinic 1, Goethe University Frankfurt, University Hospital, Frankfurt, Germany; 13 Interdisciplinary Trial Center and ECTU, University Hospital Wurzburg, Wurzburg, Germany; 14 West German Cancer Center, Department of Medical Oncology, University Hospital Essen, Essen, Germany; 15 National Center for Tumor Diseases (NCT), NCT West, Essen, Germany; 16 NEXT Oncology Phase I Unit/IOB - Hospital Quironsalud Barcelona, Barcelona, Spain; 17 Department of Dermatology, University Hospital Zurich, Zurich, Switzerland; 18 1st Medical Clinic, University Medical Center of the Johannes Gutenberg University Mainz, Main, Germany; 19 Department of Medical Oncology, Comprehensive Cancer Center, IRCCS Ospedale San Raffaele, Milan, Italy; 20 Vita Salute San Raffaele University, Milan, Italy; 21 Airway Research Center North (ARCN), German Center for Lung Research (DZL), LungenClinic, Grosshansdorf, Germany; 22 Institute of Bioinformatics, Biocenter, Medical University of Innsbruck, Innsbruck, Austria; 23 Metronomia Clinical Research GmbH, Munich, Germany; 24 Department of Medicine, Technical University Munich, Munich, Germany; 25 Department of Gynecology, University Hospital Wurzburg, Wurzburg, Germany; 26 CatalYm, Planegg-Martinsried, Germany

**Keywords:** GDF-15, Visugromab, Anti–PD-(L)1-relapsed/refractory solid tumors, Non-squamous non-small-cell lung cancer, Urothelial carcinoma, Hepatocellular carcinoma

## Abstract

**Background:**

Resistance to anti–PD-1/PD-L1 therapy is a major unmet need. Growth Differentiation Factor 15 (GDF-15) has been identified as a key resistance factor for anti–PD-1/PD-L1 immunotherapy. Visugromab, a neutralizing anti–GDF-15 antibody, plus the anti–PD-1 antibody nivolumab (V+N) was evaluated in the first-in-human phase 1/2a GDFATHER-01 trial in heavily pretreated participants with locally advanced/metastatic non-squamous non-small-cell lung cancer (nsq NSCLC), urothelial carcinoma (UC), or hepatocellular carcinoma (HCC), stringently defined as anti–PD-1/PD-L1-relapsed/refractory, and showed encouraging objective responses. This analysis reports long-term follow-up of these three phase 2 expansion cohorts of the GDFATHER-01 trial.

**Methods:**

Seventy-seven participants with nsq NSCLC (N=22), UC (N=27), and HCC (N=28) received visugromab (10 mg/kg) plus nivolumab (240 mg) every two weeks until disease progression or unacceptable toxicity.

**Results:**

Objective response rates (RECIST v1.1) were 18.2% for nsq NSCLC (4/22; 95%CI 5.2–40.3), 18.5% for UC (5/27; 95%CI 6.3–38.1), and 14.3% for HCC (4/28; 95%CI 4.0–32.7). Median duration of response (DoR) was 32.2 months (95%CI 5.5–38.0), 28.8 months (95%CI 7.4–39.4), and 19.4 months (95%CI 5.8–39.7; with protracted recruitment), respectively, with 7/13 responses (53.8%) ongoing. Confirmed complete response or complete metabolic response (CR or CMR) among responders was 61.5% (8/13), with 7/8 ongoing. In addition, 46.2% (6/13) of responders achieved a deeper response on V+N per RECIST v1.1 than with the prior anti–PD-(L)1 therapy; median DoR on V+N was 28.8 months (95%CI 7.4–38.0) versus 12.0 months (95%CI 8.0–24.0) on initial anti–PD-1/PD-L1 treatment. V+N was generally well tolerated.

**Conclusions:**

In heavily pretreated, advanced/metastatic participants with nsq NSCLC, UC, or HCC who were anti–PD-1/PD-L1-relapsed/refractory, V+N achieved deep and durable objective responses. The observed DoR, depth of response, and CR+CMR rate among responders exceeded those reported for their initial anti–PD-1/PD-L1 therapy. These findings suggest that GDF-15 blockade with visugromab can overcome resistance and enhance the magnitude and durability of anti–PD-1/PD-L1 responses, and warrant further exploration in randomized trials.

**Registry:**

ClinicalTrials.gov, TRN: NCT04725474, Registration date: 25 January 2021; EudraCT, TRN: 2020-002103-19, Registration date 16 Dec 2020

**Supplementary Information:**

The online version contains supplementary material available at https://doi.org/10.1186/s13045-026-01818-2.

## Background

Immune checkpoint inhibitors targeting PD-1/PD-L1 have transformed the therapeutic landscape of solid tumor treatment [1–3]. Yet, the vast majority of patients with advanced/metastatic solid tumors ultimately experience relapse or progression on anti–PD-1/PD-L1 therapy. Once anti–PD-1/PD-L1 relapsed/refractory, outcomes are dismal, representing a major unmet medical need [4–6].

Anti–PD-1/PD-L1 resistance appears to be mainly driven by tumor-induced immunosuppression and immune escape [4, 7]. Various approaches have been explored to overcome anti–PD-1/PD-L1 resistance, including combinations with checkpoint inhibitors targeting TIGIT, LAG-3, TIM-3, and other mechanisms [8, 9]. However, apart from possibly targeting CTLA-4 [10], no single resistance factor has been identified whose modulation results in consistent reinvigoration and potentiation of anti–PD-1/PD-L1 activity across multiple solid tumors.

Growth Differentiation Factor 15 (GDF-15) is linked to feto-maternal tolerance and tissue stress responses [11, 12]. It has been identified as the most overexpressed cytokine in solid tumors [13, 14], and its overexpression is associated with reduced overall survival in many solid tumor types. Recent investigations have revealed that GDF-15 acts as a potent local immunosuppressant by disconnecting LFA-1 from the actin cytoskeleton [15]. As a result, T-cell infiltration into tissues, particularly within the tumor microenvironment, is inhibited. LFA-1 destabilization also suppresses antigen presentation and the adaptive immune response, further contributing to an immunosuppressive tumor microenvironment [14, 15].

Visugromab (CTL-002), a humanized, hinge-stabilized, GDF-15–neutralizing IgG4 monoclonal antibody, was evaluated in combination with nivolumab in a first-in-human, phase 1/2a trial (GDFATHER-01; NCT04725474). Transcriptomic analyses of The Cancer Genome Atlas (TCGA) and various translational research datasets suggested prominent GDF-15–mediated immunosuppression in non-squamous non-small cell lung cancer (nsq NSCLC), urothelial carcinoma, and hepatocellular carcinoma (HCC) [16], directing initial clinical exploration of visugromab to these tumor types. Immune monitoring of interferon-γ-inducible chemokines supported the combination treatment concept [16]. All enrolled participants had exhausted available treatment options, including prior treatment—either as monotherapy or in combination—with an approved anti–PD-1/PD-L1 agent. Observed overall response rates (ORR) per RECIST v1.1 were encouraging, with overall good tolerability, as reported previously [16].

Here, with a 15 months later cut-off date and >90% of trial participants having completed treatment, we report mature long-term safety and efficacy results of the GDFATHER-01 trial for the three fully recruited, phase 2 expansion cohorts in nsq NSCLC, urothelial carcinoma, and HCC.

## Methods

### Trial design and participants

The GDFATHER-01 trial (NCT04725474) is a first-in-human, phase 1/2a study of the GDF-15–neutralizing antibody visugromab in combination with the anti–PD-1 antibody nivolumab. Participants were required to have either primary progression on ongoing, continuous, initial anti–PD-1/PD-L1 treatment for at least 12 weeks without a RECIST v1.1 like response (“primary refractory”) or, if an initial response had been achieved on therapy, to have relapsed while still receiving the same anti–PD-1/PD-L1 regimen (“secondary refractory”); i.e. they had to meet strict criteria for PD-1/PD-L1 refractoriness/relapse. Eligible in terms of refractoriness were hence only patients who achieved progression whilst being still on initial PD-1/PD-L1 therapy. Participants who had discontinued anti–PD-1/PD-L1 treatment for other reasons, such as intolerable adverse events (AEs) or completion of the protocol-defined maximum treatment duration, were excluded.

Apart from the required PD-1/PD-L1 refractoriness/relapse criterion, eligible participants (≥18 years , no upper age limit) were required to have ECOG performance status of 0 or 1, radiologically measurable disease at baseline and adequate organ function.

Participants with a history of ongoing immune-related adverse events (irAEs) and/or AEs of grade ≥2 that had not resolved from prior therapies, or a history or clinical evidence of CNS primary tumors or metastases, including leptomeningeal metastases, were not eligible.

In the phase 1 part, escalating doses of visugromab (0.1, 1, 3, 10, and 20 mg/kg Q2W) were administered in combination with the anti–PD-1 antibody nivolumab (240 mg Q2W), identifying 10 mg/kg Q2W as the recommended phase 2 dose for visugromab [16]. The design and results of the dose-escalation phase 1 part were described previously [16]. In the phase 2a part, expansion cohorts in various advanced/metastatic anti–PD-1/PD-L1-relapsed/refractory solid tumor types were treated at this dose level, including NSCLC (non-squamous and squamous), urothelial carcinoma, HCC, melanoma, and microsatellite-stable colorectal cancer, following Simon two-stage designs. After data review by an independent review committee, protocol-defined further expansion of the NSCLC, HCC, and urothelial carcinoma cohorts was implemented. Indication selection was supported by a large translational research program, including transcriptomic analyses of 11,000 tumor samples from TCGA, which identified nsq NSCLC and urothelial carcinoma as key GDF-15–immunosuppressed target indications [16]. Notably, for HCC, substantial GDF-15 overexpression has been reported across all major underlying liver disease conditions predisposing to HCC [17–20], suggesting a GDF-15–saturated tumor microenvironment.

The trial was approved by the national competent authorities of the participating countries and by the institutional review boards at each participating site, and has been conducted in accordance with ICH-GCP and the Declaration of Helsinki. All participants provided written informed consent. A safety review committee met regularly throughout all parts of the trial to monitor participant safety and to evaluate dose-limiting toxicities (as defined in the protocol and reported previously [16]) during phase 1.

### Trial endpoints and assessments

Primary endpoint for the phase 2a part was investigator-assessed ORR per RECIST v1.1 [21]. Additional efficacy endpoints included time-to-response (TTR), duration-of-response (DoR; measured from start of treatment until disease progression or trial discontinuation), progression-free survival (PFS), overall survival (OS), and the proportion of participants with tumor shrinkage. Tumor assessments (CT/MRI/PET) were done at 8-weekly intervals, a blinded copy of the images was sent to a central reader for monitoring purposes and post-hoc analysis. Investigators were required to use in each participant the assessment modality selected at baseline for subsequent assessments. For participants with durable partial responses (PR >12 months), FDG-PET/CT according to institutional standards was offered to assess metabolic response, including complete metabolic response (CMR).

For safety, clinical laboratory parameters, vital signs, ECGs, and physical examinations (including neurological examination) were assessed. Treatment-emergent adverse events (TEAEs) were coded using the current Medical Dictionary for Regulatory Activities (MedDRA) and graded according to the National Cancer Institute Common Terminology Criteria for Adverse Events (CTCAE), version 5.0 [22].

GDF-15 serum levels were assessed at baseline and at predefined time points throughout the trial (Quantitative solid-phase sandwich ELISA kit, manufactured by R&D Systems: GDF-15 DuoSet ELISA kit, catalogue no. DY957) [16].

### Statistical analysis

For the three main expansion cohorts (NSCLC, urothelial carcinoma, HCC) reported here, a Simon two-stage design required initial recruitment of 14 response-evaluable participants per cohort in order to allow the detection of at least one response with 80% probability if the true response rate is 20% (minimal acceptable probability of response), the maximum unacceptable response rate is 5% and the alpha level is 5% [23]. If at least one response was observed, an additional 13 participants were to be recruited, to generate cohort-wise a total of ≥4/27 responders as proof of activity for early phase 2 testing; no cohort-specific null (H_0_) and alternative (H_A_) hypotheses were defined. For ORR (and disease control rate [DCR]) analyses, summary tables were generated presenting the number and proportion of responders with two-sided 95% Clopper–Pearson confidence intervals (95%CI).

Further summary statistics were calculated for DoR, TTR, PFS, OS, and duration of treatment based on all participants with available data for the respective parameters. No formal statistical analysis of survival times was performed to model censoring in participants without an observed event.

## Results

### Participant disposition and baseline characteristics

Between July 2021 and April 2024, three expansion cohorts in advanced/metastatic NSCLC, urothelial carcinoma, and HCC each enrolled 27 participants who were relapsed/refractory to prior anti–PD-1/PD-L1 therapy as defined by stringent criteria. Participants with HCC (N=1) and NSCLC (N=2) from phase 1 dose levels 4 and 5 were added to the analysis, as these dose levels were considered therapeutically active. In total, 84 participants with NSCLC (7 squamous and 22 non-squamous), urothelial carcinoma (N=27), and HCC (N=28) were available for analysis. Thirty-five participants had received prior anti–PD-1/PD-L1 treatment as monotherapy, 20 in combination with chemotherapy, and 29 in combination with other biologics or targeted therapies. In a single patient with HCC (from phase 1), an experimental anti–PD-L1 checkpoint inhibitor (LY3300054) had been administered, which had been shown to achieve an ORR similar to that of pembrolizumab in MSI-H/dMMR solid tumors [24].

Participants had received a mean of 2.8 (95%CI 2.5–3.2) prior lines of systemic therapy (median 2.0; range 1–7). Prior anti–PD-1/PD-L1 treatment had a median duration of 7.3 months (95%CI 5.8–9.0). Median follow-up was 10.2 months (95%CI 7.6–13.5), and median treatment duration (total population) was 2.4 months (95%CI 1.4–3.3). Baseline characteristics are summarized in Table 1.

**Table 1:** Demographics and baseline disease characteristics

**Characteristic**	**NSCLC (sq.)**	**NSCLC (nsq.) **	**UC **	**HCC**	Efficacy *	**Total (safety)**
	**(n = 7)**	**(n = 22)**	**(n = 27)**	**(n = 28)**	**(n = 77)**	**(n = 84)**
Age						
Median (range) – years	65.0 (51.0-74.0)	67.5 (42.0-81.0)	67.0 (52.0-82.0)	65.5 (48.0-77.0)	67.0 (42.0-82.0)	66.0 (42.0-82.0)
Age > 65 years – no. (%)	4 (57.1)	15 (68.2)	17 (63.0)	15 (53.6)	47 (61.0)	51 (60.7)
Sex – no. (%)						
Female	1 (14.3)	6 (27.3)	5 (18.5)	6 (21.4)	17 (22.1)	18 (21.4)
Male	6 (85.7)	16 (72.7)	22 (81.5)	22 (78.6)	60 (77.9)	66 (78.6)
Race, n (%)						
White	6 (85.7)	21 (95.5)	27 (100.0)	26 (92.9)	74 (96.1)	80 (95.2)
Not reported	1 (14.3)	1 (4.5)	0	2 (7.1)	3 (3.9)	4 (4.8)
ECOG performance status – no. (%)						
0	5 (71.4)	6 (27.3)	12 (44.4)	23 (82.1)	41 (53.2)	46 (54.8)
1	2 (28.6)	16 (72.7)	15 (55.6)	5 (17.9)	36 (46.8)	38 (45.2)
Stage of cancer at enrolment – no. (%)						
Locally advanced	0	0	2 (7.4)	7 (25.0)	9 (11.7)	9 (10.7)
Metastatic	7 (100.0)	22 (100.0)	25 (92.6)	21 (75.0)	68 (88.3)	75 (89.3)
Prior cancer surgery – no. (%)						
Yes	2 (28.6)	9 (40.9)	25 (92.6)	14 (50.0)	48 (62.3)	50 (59.5)
No	5 (71.4)	13 (59.1)	2 (7.4)	14 (50.0)	29 (37.7)	34 (40.5)
Prior cancer radiotherapy – no. (%)						
Yes	6 (85.7)	18 (81.8)	15 (55.6)	7 (25.0)	40 (51.9)	46 (54.8)
No	1 (14.3)	4 (18.2)	12 (44.4)	21 (75.0)	37 (48.1)	38 (45.2)
Prior systemic therapy						
Median (range) – no.	2.0 (1-7)	3.5 (1-7)	3.0 (1-6)	2.0 (1-4)	2.0 (1-7)	2.0 (1-7)
0 (i.e., no prior therapy)	0	0	0	0	0	0
1	1 (14.3)	3 (13.6)	3 (11.1)	11 (39.3)	17 (22.1)	18 (21.4)
2	4 (57.1)	4 (18.2)	6 (22.2)	12 (42.9)	22 (28.6)	26 (31.0)
3	0	4 (18.2)	9 (33.3)	2 (7.1)	15 (19.5)	15 (17.9)
≥4	2 (28.6)	11 (50.0)	9 (33.3)	3 (10.7)	23 (29.9)	25 (29.8)
Prior platinum-based therapy – no (%)						
Yes	6 (85.7)	22 (100.0)	25 (92.6)	0 (0.0)	nr	nr
Prior Anti-PD-1/-L1 therapy – no. (%)						
Yes	7 (100.0)	22 (100.0)	27 (100.0)	28 (100.0)	77 (100.0)	84 (100.0)
Duration of initial anti-PD-1/-L1						
Median (95% CI) – months	7.0 (5.1–63.5)	6.1 (4.9– 12.0)	6.9 (3.9–10.6)	9.2 (6.9–11.2)	7.4 (5.8–9.0)	7.3 (5.8–9.0)
Anti-PD-1/-L1 therapy as most recent therapy line						
Yes	5 (71.4)	9 (40.9)	11 (40.7)	17 (60.7)	37 (48.1)	42 (50.0)
No	2 (28.6)	13 (59.1)	16 (59.3)	11 (39.3)	40 (51.9)	42 (50.0)
Number of prior anti-PD-1/-L1 therapy lines						
0 (i.e., no prior anti-PD-1/-L1	0	0	0	0	0	0
1	6 (85.7)	18 (81.8)	22 (81.5)	22 (78.6)	62 (80.5)	68 (81.0)
2	1 (14.3)	3 (13.6)	4 (14.8)	6 (21.4)	13 (16.9)	14 (16.7)
≥3	0	1 (4.5)	1 (3.7)	0	2 (2.6)	2 (2.4)

*na*, not applicable, nr = not reported * comprising HCC, UC and nsq NSCLC cohorts only

The most common reason for treatment discontinuation was disease progression (76.1%), followed by death (7.1%), adverse events (3.6%), withdrawal of consent (2.4%), and other reasons (1.2%) (Suppl. Table S1). At the current data cutoff (August 21, 2025), eight of 84 participants (9.5%) remained on treatment. Although disease control was observed, no confirmed objective responses occurred among the seven participants with squamous NSCLC (sq NSCLC). This finding, together with TCGA-based *in silico* profiling suggesting that sq NSCLC may not be predominantly GDF-15–driven, led to its exclusion from the dedicated efficacy analyses (Suppl. Fig. S1). However, given the evidence of disease stabilization and the small number of participants, clinical activity in sq NSCLC should not be excluded.

### Safety

The safety analysis set comprised 84 participants across all cohorts (squamous and non-squamous NSCLC, urothelial carcinoma, and HCC). TEAEs of any grade occurred in 83 of 84 participants (98.8%), and grade ≥3 TEAEs were reported in 39 participants (46.4%).

Treatment-related adverse events (TRAEs) were reported in 47 participants (56.0%), with grade ≥3 TRAEs occurring in 10 participants (11.9%). Three of 84 participants (3.6%) discontinued study treatment due to a TRAE (Table 2). Two grade 5 TRAEs occurred and both events were assessed as at least possibly related to visugromab and/or nivolumab (note: the latter case was reclassified as possibly related after data cutoff). One participant, a 70-year-old male with nsq NSCLC experienced multiple organ dysfunction syndrome with kidney and liver failure while on nivolumab plus visugromab. The participant had pre-existing renal vulnerability following a prior experimental antibody–drug conjugate (resolved to residual Grade 1 creatinine elevation at enrollment). One week after the first dose of visugromab plus nivolumab, he developed acute worsening renal function followed by rapid hepatic failure, both assessed as possibly related to one or both study drugs. On the day of death, after undergoing transjugular kidney and liver biopsies for diagnostic evaluation, he experienced sudden cardiac arrest; no resuscitation was attempted in accordance with a documented do-not-resuscitate order. Histology (pre-mortem biopsies and confirmatory autopsy) demonstrated a pattern overall suggestive of drug-induced renal and hepatic injury, with no evidence of malignancy. The other, a 56-year-old male also with nsq NSCLC, developed CT-confirmed pulmonary infiltrates and elevated CRP ~4 weeks after treatment initiation and was treated as respiratory infection with initial improvement. Respiratory decline recurred 2 weeks later; steroids were initiated for suspected pneumonitis in addition to adjusted antibiotics, but he progressed to Grade 5 respiratory decompensation of unclear cause. Death was attributed to respiratory insufficiency (death certificate) and no autopsy was conducted. Detailed case narratives are provided in the Supplementary Materials (pp. 5–6).

**Table 2:** Treatment-related adverse events (TRAE), visugromab plus nivolumab

**TRAE, summary (all TRAE)**	**NSCLC (sq.)**	**NSCLC (nsq.)**	**UC**	**HCC**	**Total (safety)**
	**(n = 7)**	**(n = 22)**	**(n = 27)**	**(n = 28)**	**(n = 84)**
Treatment-related Adverse events					
Any grade (Grade 1-5) – no. (%)	2 (28.6)	10 (45.5)	17 (63.0)	18 (64.3)	47 (56.0)
Grade 3-5 – no. (%)	0	4 (18.2)	3 (11.1)	3 (10.7)	10 (11.9)
Grade 5 (deaths) – no. (%)	0	2 (9.1)	0	0	2 (2.4)
Leading to discontinuation – no. (%)	0	1 (4.5)	1 (3.7)	1 (3.6)	3 (3.6)
TRAE (any grade), per MedDRA system organ class (in order of frequency)* –** no. (%)**					
General disorders	1 (14.3)	5 (22.7)	10 (37.0)	5 (17.9)	21 (25.0)
Skin & subcutaneous tissue	0	2 (9.1)	5 (18.5)	5 (17.9)	12 (14.3)
Investigations	0	3 (13.6)	3 (11.1)	5 (17.9)	11 (13.1)
Gastrointestinal	0	2 (9.1)	0	6 (21.4)	8 (9.5)
Musculoskeletal & connective tissue	0	0	3 (11.1)	5 (17.9)	8 (9.5)
Blood & lymphatic system	1 (14.3)	1 (4.5)	4 (14.8)	1 (3.6)	7 (8.3)
Metabolism & nutrition disorders	1 (14.3)	1 (4.5)	2 (7.4)	2 (7.1)	6 (7.1)
Nervous system	0	2 (9.1)	1 (3.7)	1 (3.6)	4 (4.8)
Infections & Infestations	0	2 (9.1)	0	1 (3.6)	3 (3.6)
Hepatobiliary	0	2 (9.1)	0	1 (3.6)	3 (3.6)
Respiratory, thoracic & mediastinal	0	1 (4.5)	1 (3.7)	0	2 (2.4)
Vascular	0	0	1 (3.7)	1 (3.6)	2 (2.4)
Renal & urinary	0	1 (4.5)	0	0	1 (1.2)
Cardiac	0	0	1 (3.7)	0	1 (1.2)
Endocrine	0	0	0	1 (3.6)	1 (1.2)
Immune system	0	0	1 (3.7)	0	1 (1.2)
TRAE (grade ≥3), per MedDRA Preferred Term (in order of frequency) – no. (%)					
Aspartate aminotransferase ↑	0 (0.0)	1 (4.5)	0 (0.0)	1 (3.6)	2 (2.4)
Alanine aminotransferase ↑	0 (0.0)	0 (0.0)	0 (0.0)	1 (3.6)^†^	1 (1.2)
Transaminases ↑	0 (0.0)	0 (0.0)	0 (0.0)	1 (3.6)	1 (1.2)
Cholestasis	0 (0.0)	1 (4.5)	0 (0.0)	0 (0.0)	1 (1.2)
Hepatic failure	0 (0.0)	1 (4.5)	0 (0.0)	0 (0.0)	1 (1.2)
Pneumonitis	0 (0.0)	1 (4.5)	0 (0.0)	0 (0.0)	1 (1.2)
Immune-mediated pneumonitis	0 (0.0)	0 (0.0)	1 (3.7)	0 (0.0)	1 (1.2)
Diarrhea	0 (0.0)	1 (4.5)^†^	0 (0.0)	0 (0.0)	1 (1.2)
Upper gastrointestinal hemorrhage	0 (0.0)	0 (0.0)	0 (0.0)	1 (3.6)	1 (1.2)
Hypokalemia	0 (0.0)	1 (4.5)	0 (0.0)	0 (0.0)	1 (1.2)
Decreased appetite	0 (0.0)	0 (0.0)	1 (3.7)	0 (0.0)	1 (1.2)
Pneumonia	0 (0.0)	*1 (4.5)*	0 (0.0)	0 (0.0)	1 (1.2)
Multiple organ dysfunction syndrome	0 (0.0)	*1 (4.5)*	0 (0.0)	0 (0.0)	1 (1.2)
Acute kidney injury	0 (0.0)	1 (4.5)	0 (0.0)	0 (0.0)	1 (1.2)
Hypertension	0 (0.0)	0 (0.0)	1 (3.7)^†^	0 (0.0)	1 (1.2)

* System Organ Classes (SOC) with no reported TRAE (of any grade and/or for any cohort) are not listed

† Two events (at different time points) in one patient

Values (MedDRA Preferred Terms) in italics: grade 5 events; ↑, increased

The most frequent TRAEs (≥5% of participants) of any grade included pyrexia (n=10 [11.9%]); rash in its different manifestations (n=7 [8.3%]); increased alanine aminotransferase (n=6 [7.1%]); diarrhea (n=6 [7.1%]); and asthenia and fatigue (each n=5 [6.0%]) (Suppl. Table S1). Increased aspartate aminotransferase (n=2 [2.4%]) and increased alanine aminotransferase, diarrhea, and hypertension (each n=1 [1.2%]) were the only grade ≥3 TRAEs occurring more than once (two events each) (Table 2).

In total, 176 TRAEs were reported, of which 76.1% had resolved or recovered at the time of this analysis. Among the 19 grade ≥3 TRAEs, 68.4% had resolved or recovered. TRAEs led to temporary treatment interruption in 7/84 (8.3%) participants (23 events in total) and to permanent treatment discontinuation in 3/84 (3.6%) participants (Suppl. Table S2).

### Efficacy

**Fig. 1:**
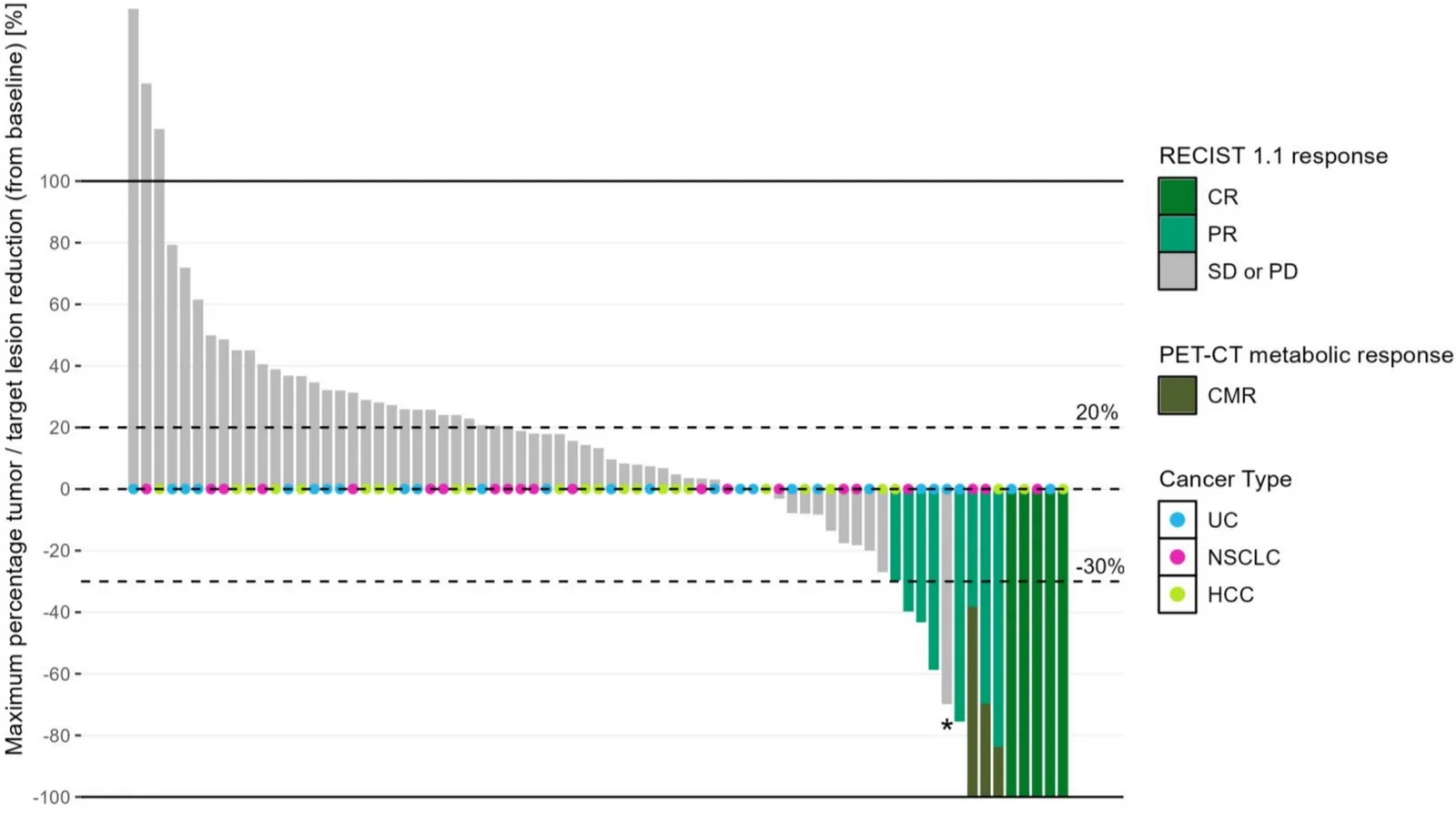
Overall response assessment: best overall response as per RECIST v1.1 and metabolic response. all participants with at least one tumor assessment according to RECIST v.1.1, i.e., currently N=73, are displayed in the waterfall Plot, indicating reductions in main target lesion (measurable disease). NSCLC, non-small cell lung cancer (non-squamous); UC, urothelial carcinoma; HCC, hepatocellular carcinoma. *PR on target lesions with simultaneous occurrence of new lesion (PD by RECIST)

Overall, the ORR was 13/77 (16.9%; 95%CI 9.3–27.1) across the three main target indications (nsq NSCLC, urothelial carcinoma, and HCC). Overall, 8/77 (10.4%) participants achieved a PR and 5/77 (6.5%) a CR as best response, with all CRs and all but one PR confirmed per RECIST v1.1. Among participants with PR, 3/8 (37.5%) achieved a CMR. The disease control rate (DCR; CR+PR+SD) was 42.9% (95%CI 31.6–54.6) (Table 3). Tumor size reduction (median −41.5%; range −3.2% to −100%) was observed in 24/77 (31.2%) participants (Fig. 1).

**Table 3:** Treatment outcomes per Indication

**Outcome**	**NSCLC (sq.)**	**NSCLC (nsq.)**		**UC**	**HCC**	**Total (efficacy)**
	**(n = 7)**		**(n = 22)**	**(n = 27)**	**(n = 28)**	**(n = 77)**
Overall response rate (ORR) – no.	0		4	5	4	13
Percentage – (95% CI)	--		18.2 (5.2–40.3)*	18.5 (6.3–38.1)	14.3 (4.0–32.7)	16.9 (9.3–27.1)
Partial response (PR) – no. (%)	0		3 (13.6)	3 (11.1)	2 (7.1)	8 (10.4)
Complete response (CR) – no. (%)	0		1 (4.5)	2 (7.4)	2 (7.1)	5 (6.5)
Complete metabolic response (CMR)						
CMR rate – no. (%)	0		2 (9.1)	0	1 (3.6)	3 (3.9)
CR or CMR (Rate, combined) – no. (%)	0		3 (13.6)	2 (7.4)	3 (10.7)	8 (10.4)
Responses ongoing (PR, CMR & CR)						
Number (%)	--		3 (75.0)	2 (40.0)	2 (50.0)	7 (9.1)
Duration of Response^†^: (DoR)						
Median (95% CI) – months	--		32.2 (5.5–38.0)	28.8 (7.4–39.4)	19.4 (5.8–39.7)	28.8 (7.4–38.0)
Time to response (TTR)						
Median (95% CI) – months	--		1.9 (1.8–5.6)	3.7 (1.9– 18.2)	3.3 (1.8–8.6)	2.8 (1.9–5.6)
Disease control rate (DCR) – no.	4		9	13	11	33
Percentage – (95% CI)	57.1 (18.4–90.1)		40.9 (20.7–63.6)	48.1 (28.7–68.1)	39.3 (21.5–59.4)	42.9 (31.6–54.6)
Progression-free survival (PFS)						
Median (95% CI) – months	3.2 (1.4–25.0)		1.8 (1.4–5.2)	2.5 (1.7–4.2)	2.0 (1.8–3.5)	1.9 (1.8–3.2)
Overall survival (OS)						
Median (95% CI) – months	14.0 (3.5–25.0)		8.8 (3.1–14.9)	11.6 (4.4–20.6)	9.8 (6.0–15.5)	10.0 (5.7–12.7)
Duration of treatment (DOT)						
Median (95% CI) – months	2.7 (1.0–7.6)		2.2 (1.4–4.6)	2.5 (1.4–6.5)	2.6 (1.4–3.7)	2.3 (1.4–3.5)
Treatment ongoing – no. (%)	0		2 (9.1)	2 (7.4)	4 (14.3)	8 (10.4)

*All but one response confirmed per RECIST v1.1; ^†^ Measured from start of treatment until progression / end of study [Am J Cancer Res 2021;11:1121-1131]

The combined complete plus complete metabolic response rate was 10.4% (8/77). All complete responses (CRs) and CMRs were confirmed. Among the 13 responders, 5 (38.5%) achieved a CR and 3 achieved a CMR, resulting in a CR or CMR rate of 8/13 (61.5%).

**Fig. 2:**
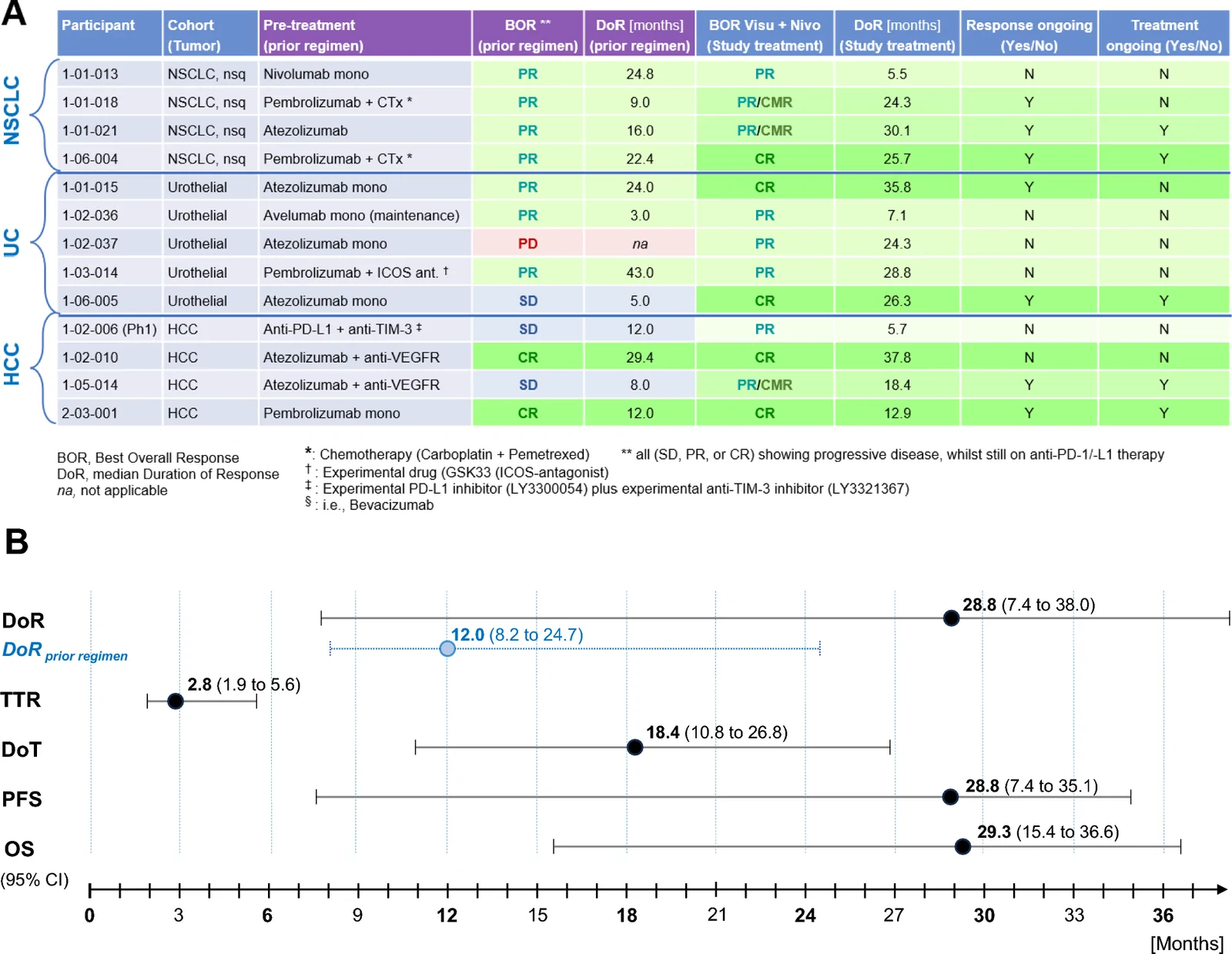
Characterization of visugromab-plus-nivolumab responders (N=13). (**A**) Comparison of response to initial anti-PD-1/-L1 treatment versus response to study treatment; (**B**) Graphical presentation of duration of response and time-to-event endpoints

Median DoR among responders was 28.8 months (95%CI 7.4–38.0), and median TTR was 2.8 months (95%CI 1.9–5.6). Responders remained on therapy for a median of 22.1 months (95%CI 10.8–30.0), with 7/13 (53.8%) responses (4 CR, 3 CMR) ongoing at the time of analysis (Fig. 2).

### Non–small cell lung cancer

Of 29 participants with NSCLC, 7 (24.1%) had squamous (sq) and 22 (75.9%) non-squamous (nsq) histology. Overall, 28/29 (96.6%) participants had received prior platinum-based chemotherapy (first-line: N=25; second-line: N=3) (Table 1), 18/28 (64.3%) of these in combination with pembrolizumab (first-line: N=11; second-line: N=5; third-line: N=1; fourth-line: N=1) as initial approved anti–PD-1/PD-L1 treatment. Other prior anti–PD-1/PD-L1 regimens included nivolumab monotherapy (second-line: N=1; third-line: N=2), pembrolizumab monotherapy (first-line: N=2; second-line: N=1), atezolizumab monotherapy (second-line: N=1; third-line: N=1; fourth-line: N=1), and nivolumab plus ipilimumab plus relatlimab (second-line: N=1). In addition, 10/29 (34.5%) participants received prior docetaxel (second or later line).

In nsq NSCLC, the ORR was 18.2% (4/22; 95%CI 5.2–40.3), with PR as best response in 13.6% (3/22) and CR in 4.5% (1/22). Two CMRs (9.1%) were observed among participants with PR, resulting in a combined CR or CMR rate of 3/22 (13.6%) in nsq NSCLC. The median DoR for nsq NSCLC at data cut-off is 32.2 months (95%CI 5.5–38.0). Median PFS was 1.8 months (95%CI 1.4–5.2) and median OS 8.8 months (95%CI 3.1–14.9) (Table 3). The DCR in nsq NSCLC was 40.9% (9/22; 95%CI 20.7–63.6), 57.1% (4/7; 95%CI 18.4–90.1) in sqNSCLC, and 44.8% (13/29; 95%CI 26.4–64.3) overall in NSCLC. No responses were observed in sqNSCLC.

**Fig. 3:**
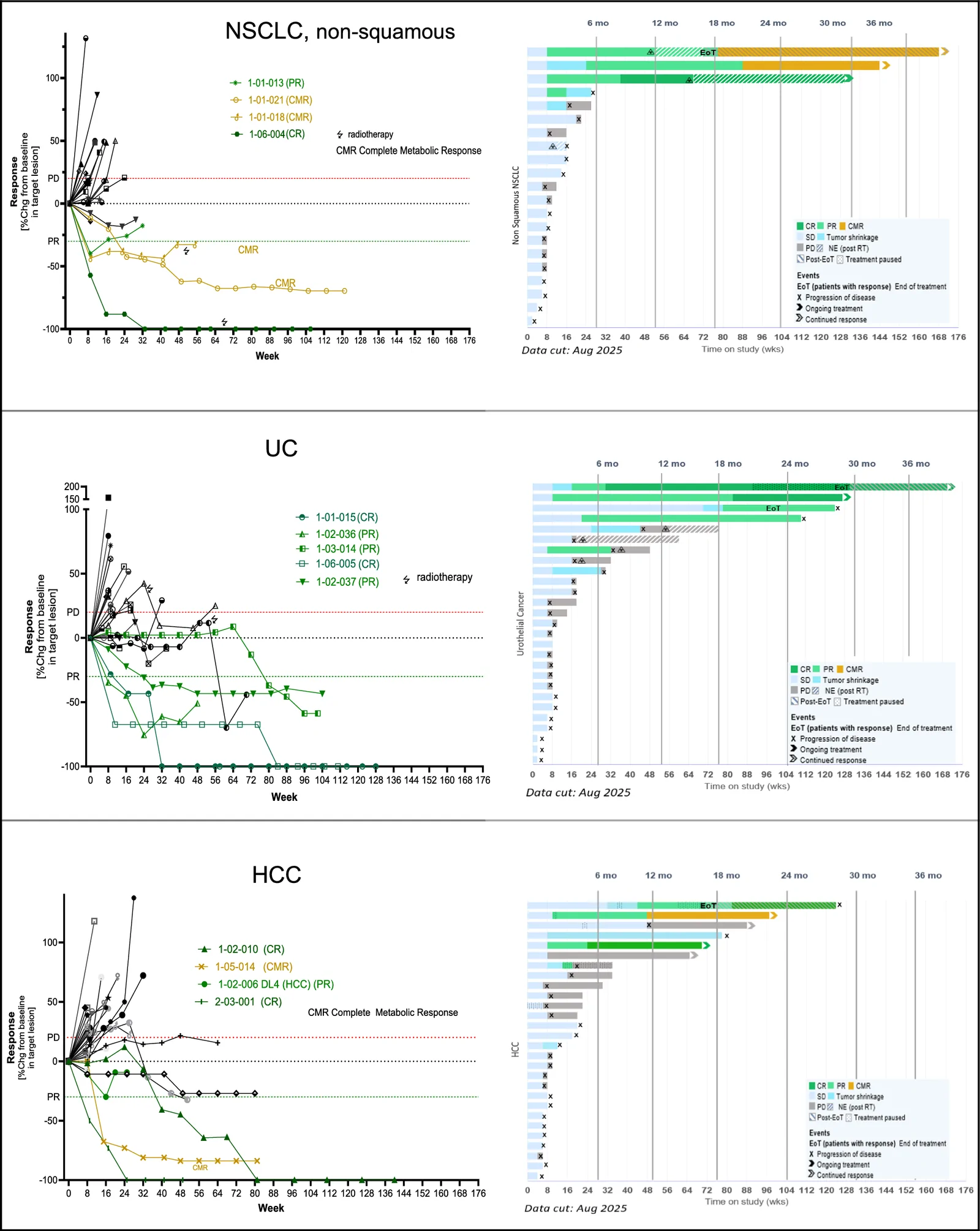
Change in tumor size (“Spider plots”) and time to and duration of response (“Swimmer Plots”). Patients with squamous cell NSCLC (N=7, no responder) were omitted from the upper graphs for the sake of clarity. Left side: Spider plots (per cancer type); right side: Swimmer plots (per cancer type). NSCLC, non-small cell lung cancer; UC, urothelial carcinoma; HCC, hepatocellular carcinoma

At data cutoff, three responses are ongoing (two CMR and one CR), and two participants remain on study treatment (Fig. 3). One participant with PR discontinued due to PD, while another discontinued study treatment due to grade 3 pneumonitis with PR ongoing at 16.0 months; CMR was achieved two months after treatment discontinuation and remains ongoing at 20.3 months. This participant had also received, per treating physician’s decision and off protocol, focal irradiation to a single lung lesion (among several bilateral lesions) after 12 months of visugromab plus nivolumab, owing to isolated enlargement not yet meeting formal PD criteria. Under continued visugromab plus nivolumab, CMR was subsequently documented after 18.0 months of treatment.

In another nsq NSCLC participant, CR was achieved after 7.0 months. At later follow-up, an FDG PET-CT (performed at the treating physician’s discretion) revealed a hypermetabolic retroperitoneal lymph node; although this did not meet RECIST v1.1 criteria for PD/relapse, stereotactic radiotherapy was administered off protocol to this region. A prior biopsy of this lymph-node region had not demonstrated malignancy. The CR in this participant is ongoing at 30.5 months (Fig. 3).

### Urothelial carcinoma

Of the urothelial carcinoma participants, 25/27 (92.6%) had received platinum-based chemotherapy, and 10 (37.0%) had received avelumab maintenance therapy as the initial prior anti–PD-1/PD-L1-containing regimen (Table 1). Other initial anti–PD-1/PD-L1-containing therapies included atezolizumab monotherapy (first-line: N=1; second-line: N=4; third-line: N=1; fourth-line: N=1), pembrolizumab monotherapy (first-line: N=1; second-line: N=1; third-line: N=3), atezolizumab plus chemotherapy (first-line: N=2), pembrolizumab plus enfortumab vedotin (first-line: N=1), pembrolizumab plus an anti-ICOS-directed antibody (third-line: N=1), and nivolumab plus trastuzumab deruxtecan (third-line: N=1).

Confirmed ORR was 18.5% (5/27; 95%CI 6.3–38.1), with PR in 3/27 (11.1%) and CR in 2/27 (7.4%); the DCR was 48.1% (13/27; 95%CI 28.7–68.1). Median DoR at data-cut-off is 28.8 months (95%CI 7.4–39.4). Median PFS was 2.5 months (95%CI 1.7–4.2) and median OS 11.6 months (95%CI 4.4–20.6) (Table 3).

At data cutoff, two responses are ongoing (two CR), and one participant remains on study treatment (Fig. 3). One participant with PR discontinued due to PD. No irradiation or other off-protocol treatments were administered in any participant with urothelial carcinoma.

### Hepatocellular carcinoma

Of the 28 participants with HCC, 22 (78.6%) had received atezolizumab plus bevacizumab as the initial anti–PD-1/PD-L1-containing regimen, 20/22 (90.9%) in the first-line setting. Other initial anti–PD-1/PD-L1-containing therapies were nivolumab monotherapy (first-line: N=2), pembrolizumab monotherapy (first-line: N=1), pembrolizumab plus lenvatinib (first-line: N=1), an investigational anti–PD-1/PD-L1 plus anti–TIM-3 antibody (third-line: N=1), and tislelizumab (first-line: N=1). In addition, 3/28 (10.7%) had received transarterial chemoembolization (TACE), and 2/28 (7.1%) radiofrequency ablation (RFA) as locoregional therapies either prior to or in conjunction with adjuvant systemic treatment.

The confirmed ORR was 14.3% (4/28; 95%CI 4.0–32.7), with PR in 7.1% (2/28), including one short-lived confirmed response at dose level 4 in phase 1, and CR in 7.1% (2/28). One durable PR was identified as a CMR, resulting in a combined CR or CMR rate of 3/28 (10.7%) (Table 3). The DCR was 39.3% (11/28; 95%CI 21.5–59.4). Median DoR is 19.4 months (95%CI 5.8–39.7) at the time of this analysis, reflecting the more protracted recruitment of the HCC cohort compared with the NSCLC and urothelial carcinoma cohorts. Median PFS was 2.0 months (95%CI 1.8–3.5) and median OS 9.8 months (95%CI 6.0–15.5).

At data cutoff, two responses are ongoing (one PR/CMR and one CR), and two participants remained on study treatment. One participant with an ongoing CR discontinued treatment in CR after 27.9 months because of a persistent grade 2 pruritus considered immune-mediated. Another participant discontinued treatment in PR after 15.0 months due to a persistent grade 2 arthritis, also considered immune-mediated. Notably, CR was detected two months after treatment discontinuation and persisted for 23.8 months off treatment until disease progression was observed (Fig. 3). No irradiation or other off-protocol treatments were administered in any participant with HCC.

### Comparing visugromab plus nivolumab response versus response to prior anti-PD1/-L1 treatment

Of the 13 participants with a response, 6 (46.2%) achieved a deeper RECIST v1.1 response level compared with their best overall response to initial approved anti–PD-1/PD-L1 treatment (Fig. 2)**.** When CMR is included as an additional marker of response quality, 8/13 (61.5%) responders achieved an improved, deeper response level (with limitation of no information on metabolic activity during PR on prior anti–PD-1/PD-L1 treatment). All remaining responders achieved at least the same response level as with prior anti–PD-1/PD-L1 therapy. Median DoR was 28.8 months with visugromab plus nivolumab versus 12.0 months on initial anti–PD-1/PD-L1 treatment (Fig. 2).

Exemplary case narratives for responders are provided in the Supplementary Materials (page 7 ff).

## Discussion

Here we report the long-term follow-up of the first-in-human phase 1/2a trial of visugromab (GDFATHER-01), with a main emphasis on the three expansion cohorts in NSCLC, urothelial carcinoma, and HCC. Extensive translational research investigations, including high-throughput immune-transcriptomic analyses, had identified these tumor types (among others) as likely to be GDF-15–immunosuppressed.

Strict criteria for defining anti–PD-1/PD-L1 relapsed/refractory status were applied to ensure that retreatment with an anti–PD-1/PD-L1 antibody alone (here nivolumab) would not be expected to yield substantial antitumor activity. Multiple retrospective studies in similarly stringently defined anti–PD-1/PD-L1 relapsed/refractory populations have shown that response rates remain in the range of 0–5% with anti–PD-1/PD-L1 retreatment as monotherapy where relapse/progression had to have occurred during ongoing anti–PD-1/PD-L1 treatment [25–30], as in our trial.

This first-in-human trial of visugromab had previously demonstrated that GDF-15 neutralization can reinduce sensitivity to anti–PD-1/PD-L1 treatment [16]. The long-term follow-up data presented here now provide evidence that GDF-15 neutralization may also enable substantially deeper and more durable remissions than those achieved with initial anti–PD-1/PD-L1 therapy.

Notably, visugromab plus nivolumab showed an acceptable safety and tolerability profile. Retrospective studies investigating retreatment of patients with anti-PD-1/L1 therapy reported rates of higher grade irAEs in the range of 13-30% in NSCLC, HCC or across multiple tumor types [31–33]. The 11.9% rate of grade ≥3 TRAE in our combination therapy study reflects these experiences that retreatment not only may reinduce responses but also known or new irAE, even of higher grade. However, the two possibly treatment-related deaths show that caution is needed in all patients exposed to such immunotherapy. The adverse event profile of visugromab plus nivolumab largely resembled that of anti–PD-1/PD-L1 monotherapy. To note that the GDFATHER-01 population may reflect individuals who previously tolerated anti–PD-1/PD-L1 therapy, as those with serious immune-mediated adverse events during prior exposure were excluded. Additionally, patients treated at earlier disease stages often remain on therapy for extended periods, which can increase the likelihood of late-onset irAEs.

As general limitations of our dataset, the relatively small number of participants analyzed for efficacy (N=77) and the absence of randomized controlled data must be acknowledged. The total number of responders, the depth and duration of responses compared with their responses to initial anti–PD-1/PD-L1 therapy (serving here as an internal benchmark), and the observation of this effect across three major solid tumor types nonetheless provide some confidence in the observed therapeutic signal. However, randomized controlled trials will be required to confirm and more precisely define the effect size achievable with GDF-15 blockade in combination with anti–PD-1/PD-L1 therapy in different clinical settings. Ongoing biomarker analyses of this and other ongoing trials will need to further decipher the potential responder population for GDF-15 blockade and the role of GDF-15 as a predictive marker or factor for enrichment strategies.

With regard to DoR and response depth in comparable PD-1/PD-L1–relapsed/refractory populations, the results with visugromab plus nivolumab appear encouraging when benchmarked against other current immunotherapeutic combination concepts or standard-of-care options. Overall, the DoR and CR+CMR rates achieved with visugromab plus nivolumab in nsq NSCLC, urothelial carcinoma, and HCC can be considered clinically meaningful (see comprehensive literature analysis and detailed comparative benchmarking data in Additional Materials). In advanced/metastatic nsq NSCLC, approved second-line therapies (docetaxel or docetaxel plus nintedanib) achieve ORRs of 3.6–14% [34], and docetaxel plus ramucirumab yields ORRs of 23–34% [35]. However, CRs with these regimens are rare, and DoR generally remains poor at <6 months. Experimental immunotherapy combinations have produced ORRs in the range of ≈5–20%, again with CRs being virtually absent, DoR of 2.0–12.9 months, PFS of 2.1–5.6 months, and OS of 6.2–17.4 months. Other treatment approaches, such as ADCs, can yield higher ORRs but are similarly characterized by limited durability, with DoR of 6.7–11.6 months, PFS of 2.7–6.4 months, and OS of 8.8–14.6 months. Comparable patterns are seen in benchmarking analyses for urothelial carcinoma and HCC in the anti–PD-1/PD-L1 relapsed/refractory setting (see Additional Materials).

Initial biomarker analyses from this trial have been reported previously. Various factors were evaluated, but no single marker has yet been identified that reliably predicts response [16]. Of note, responses were observed independent of tumoral PD-L1 status. Potentially predictive biomarker candidates identified through tumor and blood transcriptional analyses will be investigated in more detail in currently ongoing randomized clinical trials.

Beyond its tumor-immunosuppressive effect, GDF-15 has recently gained major attention as a mediator of anorexia, nausea, and cancer cachexia [36, 37]. These metabolic effects are mediated via the GFRAL receptor in the brainstem and may occur independently of the immune-mediated effects of GDF-15. The anti–GDF-15 antibody ponsegromab has successfully completed a phase 2 trial, demonstrating weight gain and increased activity in a manifestly cachectic cancer population with elevated GDF-15 [38, 39]. Phase 3 development is ongoing. In our trial, the majority of participants did not have manifest cachexia, and no weight gain was observed in non-cachectic individuals. However, the small subset of participants who were cachectic at baseline appeared to experience a comparable degree of weight gain [16]. This dual effect may be synergistic: effective control of cancer cachexia may allow affected or at-risk patients to remain on therapy for prolonged periods, during which the antitumor activity of GDF-15 blockade combined with anti–PD-1/PD-L1 treatment can fully unfold. It is also known that platinum compounds [40], ADCs [41], and kinase inhibitors induce GDF-15, often to substantial and sustained levels. Thus, GDF-15 blockade may act on multiple fronts, preventing nausea, emesis, and cachexia, as well as reversing immunosuppression that would otherwise attenuate or suppress the antitumoral activity of anti–PD-1/PD-L1 therapy.

GDF-15 neutralization by visugromab resulted in resensitization to and enhancement of anti–PD-1/PD-L1 activity in a subset of patients with advanced/metastatic nsq NSCLC, urothelial carcinoma, and HCC who were, by strict criteria, anti–PD-1/PD-L1 relapsed/refractory and had exhausted available treatment options. Median DoR among responders across these tumor types exceeded 24 months, and a notable number of CRs and CMRs were achieved. These findings are unprecedented in comparable patient populations retreated with checkpoint inhibitors or other immunotherapies and suggest that GDF-15 blockade may both reinvigorate and substantially enhance the magnitude and durability of anti–PD-1/PD-L1 therapy.

Recently, initial results from the GDFATHER-Neo trial (NCT06059547), which compares visugromab plus nivolumab versus placebo plus nivolumab in neoadjuvant muscle-invasive bladder cancer, were reported [42]. In this small (N=30), blinded, placebo-controlled, biomarker-oriented phase 2 trial, addition of visugromab to nivolumab in a checkpoint inhibitor–naïve setting resulted in a threefold increase in pathologic and radiologic response rates compared with anti–PD-1 monotherapy. Based on these encouraging data, further investigations in anti–PD-1/PD-L1–naïve populations, including nsq NSCLC, are ongoing.

In summary, our findings may open a new avenue for the treatment of anti–PD-1/PD-L1 relapsed/refractory advanced/metastatic solid tumors, with an overall favorable tolerability profile and as a strategy with the potential to substantially potentiate checkpoint inhibitor activity. Ongoing and future randomized controlled trials in earlier lines of therapy, particularly in anti–PD-1/PD-L1–naïve populations, will be crucial to define the full therapeutic potential of GDF-15 neutralization in these tumor types.

## Supplementary Information


Additional file 1.


## Data Availability

Aggregated, more detailed trial data will be provided later – in alignment with the European Union rules for conducting clinical trials (Regulation EU No. 536/2014 of 16 April 2024) and the rules for the registration and transparent reporting of clinical trials in WHO Primary Registers – in form of a full Clinical Study Report. Additional information is provided inside the Supplement Materials published with the original article. In addition, interested researchers may submit a substantiated request for access to data to the trial sponsor (info@catalym.com)
