# Supplementary material for: Long-term follow-up of a phase 1/2 trial of anti-GDF-15 antibody visugromab plus anti-PD-1 antibody nivolumab in anti-PD-1/-L1 relapsed/refractory solid tumors
Source: J Hematol Oncol. 2026 Jul 9;19:64. doi: 10.1186/s13045-026-01818-2 (PMC13536637; doi:10.1186/s13045-026-01818-2)
Supplement: Supplementary file 1 [file 13045_2026_1818_MOESM1_ESM.docx]

**Supplementary Materials**

Supplement to: Ignacio Melero, *et al*. ***Long-term follow-up of a phase 1/2 trial of anti-GDF-15 antibody visugromab plus anti-PD-1 antibody nivolumab in anti-PD-1/-L1 relapsed/refractory solid tumors***

**Content**

| Figure S.1: Trial design phase 1/2a GDFATHER trial (Flow chart) | Page 2 |
| --- | --- |
|  |  |
| Table S.1: Treatment-Related Adverse Events (any grade), per MedDRA preferred term | Page 3 |
| Table S.2: Reasons for Treatment Discontinuation | Page 4 |
|  |  |
| Safety - Patient narrative 3-02-003: 70 years old male, nsq NSCLC (grade 5 TRAE) | Page 5 |
| Safety - Patient narrative 2-01-006: 56 years old male, nsq NSCLC (grade 5 TRAE) | Page 7 |
| Efficacy - Patient narrative 1-01-021: 60 years old male, nsq NSCLC (responder; PR + CMR) | Page 8 |
| Efficacy - Patient narrative 1-06-005: 70 years old female, Urothelial Carcinoma (responder, CR) | Page 10 |
| Efficacy - Patient narrative 2-03-001: 68 years old female, HCC (responder, CR) | Page 12 |
|  |  |
| Protocol | Page 14 |

**Figure S.1: Flow- chart: trial design of phase 1/2a GDFather-01 trial (**[NCT04725474](https://clinicaltrials.gov/study/NCT04725474)**)**

**Table S.1: Treatment-Related Adverse Events (any grade), per MedDRA Preferred Term, in more than 5% of participants in any cohort (in order of frequency)^†^** – **no. (%)**

|  | **NSCLC sq** | **NSCLC nsq** | **UC** | **HCC** | **Total (Safety)** |
| --- | --- | --- | --- | --- | --- |
| **SOC and PT** | **(n = 7)** | **(n = 22)** | **(n = 27)** | **(n = 28)** | **(n = 84)** |
| Pyrexia | 0 | 2 (9.1) | 5 (18.5) | 3 (10.7) | 10 (11.9) |
| Rash (various manifestations) | 0 | 1 (4.5) | 2 (7.4) | 4 (14.3) | 7 (8.3) |
| Diarrhoea | 0 | 2 (9.1) | 0 | 4 (14.3) | 6 (7.1) |
| Alanine aminotransferase ↑ | 0 | 1 (4.5) | 1 (3.7) | 4 (14.3) | 6 (7.1) |
| Asthenia | 0 | 1 (4.5) | 2 (7.4) | 2 (7.1) | 5 (6.0) |
| Fatigue | 0 | 1 (4.5) | 3 (11.1) | 1 (3.6) | 5 (6.0) |
| Pruritus | 0 | 0 | 3 (11.1) | 1 (3.6) | 4 (4.8) |
| Aspartate aminotransferase ↑ | 0 | 1 (4.5) | 0 | 3 (10.7) | 4 (4.8) |
| ɣ-Glutamyl transferase ↑ | 0 | 2 (9.1) | 0 | 2 (7.1) | 4 (4.8) |
| Blood alkaline phosphatase ↑ | 0 | 1 (4.5) | 0 | 2 (7.1) | 3 (3.6) |
| Anaemia | 1 (14.3) | 0 | 2 (7.4) | 0 | 3 (3.6) |
| Arthralgia | 0 | 0 | 2 (7.4) | 1 (3.6) | 3 (3.6) |
| Headache | 0 | 2 (9.1) | 1 (3.7) | 0 | 3 (3.6) |
| Abdominal pain | 0 | 0 | 0 | 2 (7.1) | 2 (2.4) |
| Decreased appetite | 1 (14.3) | 0 | 1 (3.7) | 0 | 2 (2.4) |
| Blood lactate dehydrogenase ↑ | 0 | 2 (9.1) | 0 | 0 | 2 (2.4) |
| Oedema, peripheral | 1 (14.3) | 0 | 0 | 0 | 1 (1.2) |

HCC, hepatocellular carcinoma; NSCLC, non-small cell lung carcinoma; UC, urothelial carcinoma; nsq, non-squamous; sq, squamous; 🡩, increased.

† Preferred Terms (PT) not observed in at least 5% of participants (of any grade and/or in any of the cohorts) are not listed

**Table S.2:** **Reasons for Permanent Treatment Discontinuation (for any reason)**

|  | **NSCLC sq** | **NSCLC N-sq** | **UC** | **HCC** | **Total (Safety)** |
| --- | --- | --- | --- | --- | --- |
| **Reason** | **(n = 7)** | **(n = 22)** | **(n = 27)** | **(n = 28)** | **(n = 84)** |
| **No. of patients discontinuing treatment (%)** | 7 (100.0) | 20 (90.9) | 25 (92.6) | 24 (85.7) | 76 (90.5) |
| AE not related to disease progression | 0 | 1 (4.5) | 1 (3.7) | 1 (3.6) | 3 (3.6) |
| Lost to Follow-Up | 0 | 0 | 0 | 0 | 0 |
| Objective Disease Progression | 6 (85.7) | 16 (72.7) | 17 (63.0) | 19 (67.9) | 58 (69.0) |
| Protocol Deviation | 0 | 0 | 0 | 0 | 0 |
| Subject Non-compliance | 0 | 0 | 0 | 0 | 0 |
| Sponsor Decision | 0 | 0 | 0 | 0 | 0 |
| Physician Decision | 0 | 0 | 0 | 0 | 0 |
| Treatment Withdrawal by Subject | 1 (14.3) | 0 | 0 | 0 | 1 (1.2) |
| Study Withdrawal by Subject | 0 | 0 | 1 (3.7) | 0 | 1 (1.2) |
| Unacceptable Toxicity | 0 | 0 | 0 | 0 | 0 |
| Death | 0 | 2 (9.1) | 1 (3.7) | 3 (10.7) | 6 (7.1) |
| Death due to COVID-19 | 0 | 0 | 0 | 0 | 0 |
| Subject diagnosed with COVID-19 | 0 | 0 | 0 | 0 | 0 |
| COVID-19 Subject fears | 0 | 0 | 0 | 0 | 0 |
| COVID-19 Movement restrictions | 0 | 0 | 0 | 0 | 0 |
| COVID-19 Sponsor action | 0 | 0 | 0 | 0 | 0 |
| Hospital restricted access to clinic (COVID-19) | 0 | 0 | 0 | 0 | 0 |
| Chaperone unavailable due to COVID-19 | 0 | 0 | 0 | 0 | 0 |
| Study cancelled due to COVID-19 | 0 | 0 | 0 | 0 | 0 |
| Clinical/Symptomatic Disease Progression | 0 | 1 (4.5) | 4 (14.8) | 1 (3.6) | 6 (7.1) |
| Other | 0) | 0 | 1 (3.7) | 0 | 1 (1.2) |

**Safety – Participant narrative 3-02-003: 70 years old male, nsq NSCLC (Grade 5 TRAE)**

The participant with multiple organ dysfunction syndrome had received prior treatment with an experimental antibody drug conjugate until 30 days prior to trial entry for NSCLC treatment that had resulted in kidney toxicity. The AE had resolved to residual Grade 1 creatinine elevation that allowed for trial inclusion. One week after the first administration of visugromab and nivolumab, impairment of kidney function occurred followed by rapid hepatic failure, both evaluated as possibly related to one or both study drugs (visugromab and/or nivolumab). One hour after a diagnostic transjugular kidney and liver biopsy sudden cardiac arrest occurred while the patient was hospitalized and attached to a monitor, but no resuscitation was initiated due to an existing patient-signed “do-not-resuscitate” declaration on file with the hospital.

*Summary of histologic findings in participant 3-02-003*

The autopsy showed an inhomogeneous coloration of the kidney as an expression of acute renal damage. A diagnostic kidney transjugular biopsy performed pre-mortem on the day of death had revealed renal cortical and medullary tissue with up to 10 glomeruli in part with slightly increased cellularity, consistent capillary loops and one glomerulus with capsular fibrosis. Focal mixed cell inflammatory infiltrate including numerous eosinophil granulocyte and edema in the interstitium with widespread acute tubular necrosis was noted. The intrarenal arteries had slight intimal fibrosis and tubules were dilated. Further examinations revealed acute damage to the renal tubules (tubular necrosis) with adjacent microabscesses and reactive cell changes. All the histological findings were consistent with an acute interstitial nephritis, compatible with a drug-toxic renal injury however no granulomas, fibrosis, significant tubulitis, and vasculitis were noted. The unusual widespread acute tubular necrosis without significant tubulitis confirmed the acute tubular injury could be due to an additional cause. Immunofluorescence microscopy revealed renal tissue with up to 7 glomeruli, one of which completely sclerosed, factor VIII and fibrinogen in normal patterns, and IgG, IgM, IgA, complement factors C1q and C3 all negative. The pre-mortem liver transjugular biopsy (also performed on the day of death) revealed regular architecture and more than 15 portal tracts in which mixed cell inflammatory infiltrate including numerous eosinophil granulocytes, interface hepatitis, signs of bile duct damage and ductular reaction (CK19 staining) were observed. There were no portal fibrosis and no fibrous septa. The lobular parenchyma was noted with several necroinflammatory lesions with numerous granulocytes, individual cell necrosis, Kupffer cell siderosis and hepatocellular cholestasis. No steatosis and no PAS positive, diastase resistant glomerular inclusions were noted, and consistent central veins were present.  There was no evidence of malignancy. In summary, acute kidney and hepatic cell damage was detected. Overall kidney and hepatic histologic findings were suggestive of drug induced injury. The investigations from the main autopsy confirmed the findings of the pre-mortem biopsies with no additional or diverging findings.

**Safety – Participant narrative 2-01-006:  56 years old male, nsq NSCLC (Grade 5 TRAE)**

The second participant with Grade 5 outcome (56 years old, male) was diagnosed with NSCLC, too. The patient experienced Grade 2 respiratory infection of unknown origin with CRP increase and pulmonary infiltrates (confirmed by CT) four weeks after initiation of study treatment. Under antibiotic treatment, CRP decreased, and the patient also improved clinically. However, 2 weeks after diagnosis of the respiratory infection, the patient´s respiratory condition worsened. Steroid treatment was added and antibiotics adjusted, as pneumonitis was suspected on top of infection. Finally, the participant experienced a Grade 5 respiratory decompensation while the exact cause of respiratory decompensation remained unclear. A low-dose CT control scan had showed worsening of pneumonic infiltration as well as enlarged mediastinal lymph nodes (due to infection/differential diagnosis of tumor progression). The cause of death, per death certificate, was respiratory insufficiency and no autopsy was conducted.

**Efficacy –** **Participant narrative 1-01-021: 60 years old male, nsq NSCLC (PR + CMR)**

Participant 1-01-021 is a 60 years old male diagnosed with non-squamous non-small cell lung cancer (NSCLC; stage IV [TNM _unknown_]) in December 2018. Information on tumor mutational and PD-L1 status is not available.

The participants medical history included hypercholesterolemia (Grade 1), back pain (Grade 1), and Chilaiditi syndrome (Grade 1), all ongoing at the time of informed consent.

Prior NSCLC treatments included cisplatin and pemetrexed (14-Jan-2019 to 26-Mar-2019) followed by pemetrexed maintenance (14-Jun-2019 to 06-Aug-2019), atezolizumab monotherapy (Sep-2019 to Jan-2021) with best response of partial response (PR), followed by progressive disease (PD) detected Jan-2021 on ongoing treatment, docetaxel (Feb-2021 to 2022), and carboplatin plus vinorelbine (18-Apr-2022 to 08-Jul-2022). PD was confirmed at the end of each above therapy. In total, five lines of prior systemic therapy had been administered. In addition, palliative radiotherapy to the left lung was performed in November 2021.

The participant signed informed consent for study participation on 20-Oct-2022 under protocol GlobalVersionV5.0 and was enrolled in Part B of the trial into the NSCLC cohort. At study entry, the ECOG performance status was 1. The disease stage was IV (metastatic disease).

In baseline imaging, target lesions in the right and left adrenal gland as well as in the right lung were identified with a total sum in diameters of 74 mm. No non-target lesions were identified.

The first dose of visugromab (10 mg/kg Q2W) and nivolumab (240 mg Q2W) was administered on 26-Oct-2022.

In the first response assessment (post cycle 4), tumor shrinkage (-11.5%) had started and evolved to PR (-42.6%) as per RECIST 1.1 after cycle 12 (12-Apr-2022). Further reduction was noted after cycle 24 (-62.2%; 27-Sep-2023). Duration of response is 33.9 months with ongoing PR (as of 21-Aug-2025). To evaluate tumor viability, a PET-CT was performed on 22-May-2024 and demonstrated complete metabolic response (CMR) with no signal from the remaining lesion. Further PET-CT from 03-Jul-2024, 28-Aug-2024 and 22-Oct-2024 confirmed continuation of the CMR. The participant remains on study treatment (as of 21-Aug-2025) with a total duration of treatment of 33.9 months.

Treatment-related adverse event of lactate dehydrogenase increase (Grade 1) was reported.

In summary, participant 1-01-021 received visugromab and nivolumab for 33.9 months so far, and achieved a PR (-69.6%; 12-Apr-2022), identified as confirmed complete metabolic response by sequential PET-CTs during study treatment (22-May-2024 and thereafter). Study treatment continues and is well tolerated, with CMR ongoing as of 21-Aug-2025.

**Participant 1-01-021 (NSCLC): Computer tomography (CT) and CT-PET Imaging**

**Efficacy – Participant narrative 1-06-005: 70 years old female, PD-1/-L1 refractory, with UC (CR)**

Participant 1-06-005 is a 70-yo female diagnosed with urothelial carcinoma (UC) of the distal ureter (UC; stage I, T_2_N_0_M_X_) in June 2020. Information on tumor-mutational and the PD-L1 status at baseline indicated a TPS of 10% and CPS of 50.

Medical history included hypertension (Grade 3, ongoing), dyslipidaemia, cataracts, haemorrhoids, and a history of radical hysterectomy for leiomyoma, and deep vein thrombosis (bilateral upper limbs; November 2020).

Initially, primary resection of the left distal ureter with lymphadenectomy was performed in Jul-2020. Initial systemic adjuvant therapy consisted of cisplatin (later carboplatin due to toxicity) combined with gemcitabine (Sep-2020 to Dec-2020). After progression to metastatic disease, atezolizumab was subsequently initiated in August 2022 with best response of stable disease (SD) but was discontinued in December 2022 due to detection of progression (PD). In total, two lines of prior systemic therapy had been administered.

The participant signed informed consent for study participation on 30-Jan-2023 under protocol version GlobalVersion5.0 and was enrolled in Part B of the study into the Urothelial Carcinoma cohort. At study entry, the ECOG performance status was 0. The disease stage was IV (metastatic disease).

In baseline imaging, a pelvic tumor mass and retroperitoneal iliac lymph nodes were identified as target lesions with a total sum of diameters of 89 mm. Pulmonary micronodules in both upper lobes of the lung were identified as non-target lesions.

The first dose of visugromab (10 mg/kg Q2W) and nivolumab (240 mg Q2W) was administered on 20-Feb-2023. In the first response assessment (post cycle 4; 17-Apr-2023), a PR (-67.4%) was detected and confirmed in subsequent assessments. After cycle 38 (13-Sep-2024) a complete response (CR) was detected, confirmed also by negative PET-CT. CR was maintained and confirmed in several subsequent scans (as of 21-Aug-2025). The duration of response and duration of treatment is at present 30.0 months, with study treatment ongoing as of 21-Aug-2025.

No treatment-related adverse events have been reported yet.

In summary, participant 1-06-005 receives visugromab and nivolumab for 30.0 months, initially achieving a PR, followed by CR 18.0 months after start of study treatment (cycle 38 onwards). The CR is ongoing for 11.4 months as of 21-Aug-2025 and the participant remains on study treatment. The patient maintains an ECOG performance status of 0, leads a normal daily life, and exhibits no signs of toxicity.

**Participant 1-06-005 (Urothelial Carcinoma): Computer tomography (CT)**

**Efficacy – Participant narrative 2-03-001: 68 years old female with HCC, PD-1/L1 relapsed (CR)**

Participant 2-03-001 is a 68-years old female diagnosed with hepatocellular carcinoma (HCC; stage I, T2N0M0) in January 2019. Information on tumor-mutational are not available and PD-L1 status at baseline indicated a TPS of 3% and a CPS of 12.

Medical history included obesity (Grade 2; since 1982), hypothyroidism (Grade 1; since 1999), arterial hypertension (Grade 2; since 2016), liver fibrosis (Grade 2, since 2018), Covid-19 infection from 01-Mar-2022 to 12-Mar-2022, pulmonary embolism from May 2023 to 20-Jul-2023 and periodontitis (Grade 2; since Feb-2024).

Initial tumor resection was performed 03-Jan-2019, and a subsequent resection on December 2020. Prior systemic HCC treatment was initiated in context of a clinical trial (IMMULAB phase 2) with pembrolizumab in non-metastatic state on 19-Jul-2021 combined with microwave ablation (from 31-Aug-2021 to 28-Sep-2021), all with best response of complete response (CR) before disease progression occurred 19-Jul-2022 on ongoing pembrolizumab, resulting in termination of this treatment. Another local tumor resection was performed (August 2022). Thereafter, tumor progression occurred involving the peritoneum (T4 stage), leading to administration of atezolizumab and bevacizumab from 15-Nov-2022 to 07-Feb-2023 with best response of progressive disease (PD), including now newly detected metastases in the lung. Subsequently, cabozantinib was administered from 02-Mar-2023 to 12-Oct-2023 with best response of stable disease (SD), and after progression on cabozantinib, sorafenib from 12-Oct-2023 to 12-Mar-2024 with best response of SD before disease progression was detected again 02-Jan-2024. One-time TACE was performed on 15-Feb-2024. In total, four lines of prior systemic therapy had been administered.

The participant signed informed consent for participation in the trial on 18-Mar-2024 under protocol version Version6.0_DE and was enrolled in Part B of the trial into the Hepatocellular Cancer cohort. At entry, the ECOG performance status was 0. The disease stage was IV with multiple lung metastases and multiple liver lesions). In baseline imaging, lung lesions in the left and right lobe and a lesion in liver segment II/III were identified as the target lesions with a total sum of diameters of 56 mm at baseline. Disseminated liver lesions and lung metastases in both lungs were identified as non-target lesions.

The first dose of visugromab (10 mg/kg Q2W) and nivolumab (240 mg Q2W) was administered on 04-Apr-2024. Partial response (-50.0%) per RECIST 1.1. was detected at the first response assessment (post cycle 4; 28-May-2024) and confirmed in the subsequent re-staging with reduction of -73.2% (25-Jul-2024). In the subsequent response assessment (19-Sep-2024), CR was reported for the first time and confirmed subsequently, including PET-CT with no metabolic activity detected in prior tumor areas. The duration of response is 16.5 months. The participant received study treatment for a total of 16.5 months so far and continues on treatment with ongoing CR (as of 21-Aug-2025).

Treatment-related adverse events of exanthema (Grade 1 and Grade 2) were reported.

In summary, participant 2-03-001 received visugromab and nivolumab so far for 16.5 months, having achieved a complete response 5.5 months after initiation of study treatment, and remains on treatment with ongoing, confirmed CR as of 21-Aug-2025.

**Participant 2-03-001 (Hepatocellular Carcinoma): Computer tomography (CT) and CT-PET Imaging**

**Study protocol**

The study protocol has been published previously (i.e. as ‘Supplementary information’ of the initial publication in 2025 in *Nature* (Melero I *et al*, doi: 10.1038/s41586-024-08305-z, PMID: 39663448) reporting phase 1 and interim phase 2a data) and can be accessed via following URL:

<https://static-content.springer.com/esm/art%3A10.1038%2Fs41586-024-08305-z/MediaObjects/41586_2024_8305_MOESM3_ESM.pdf>
